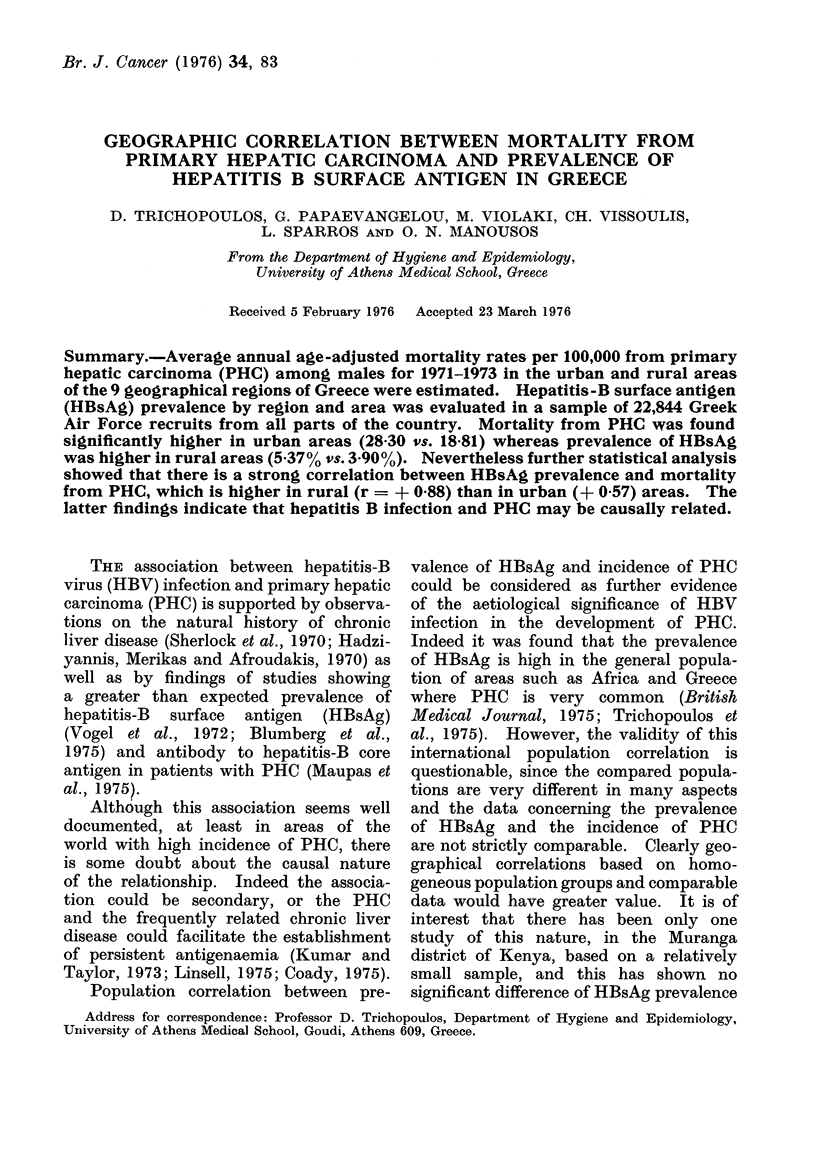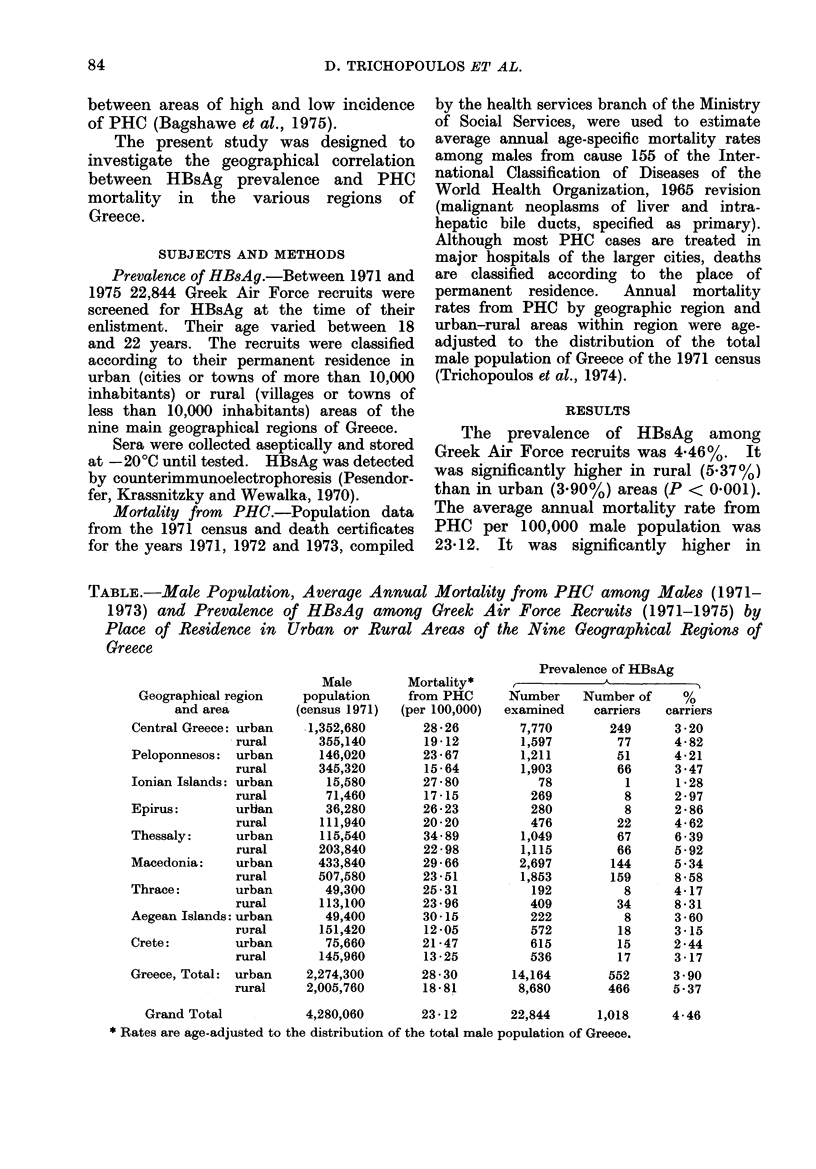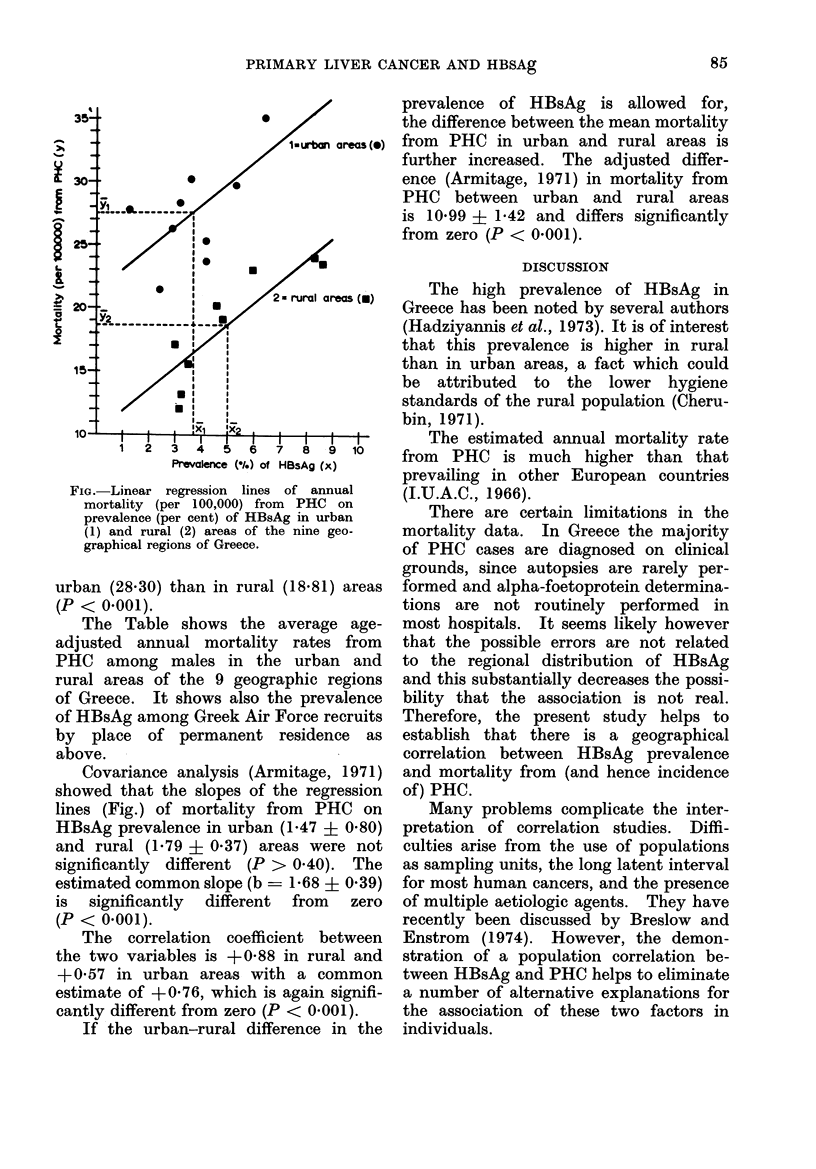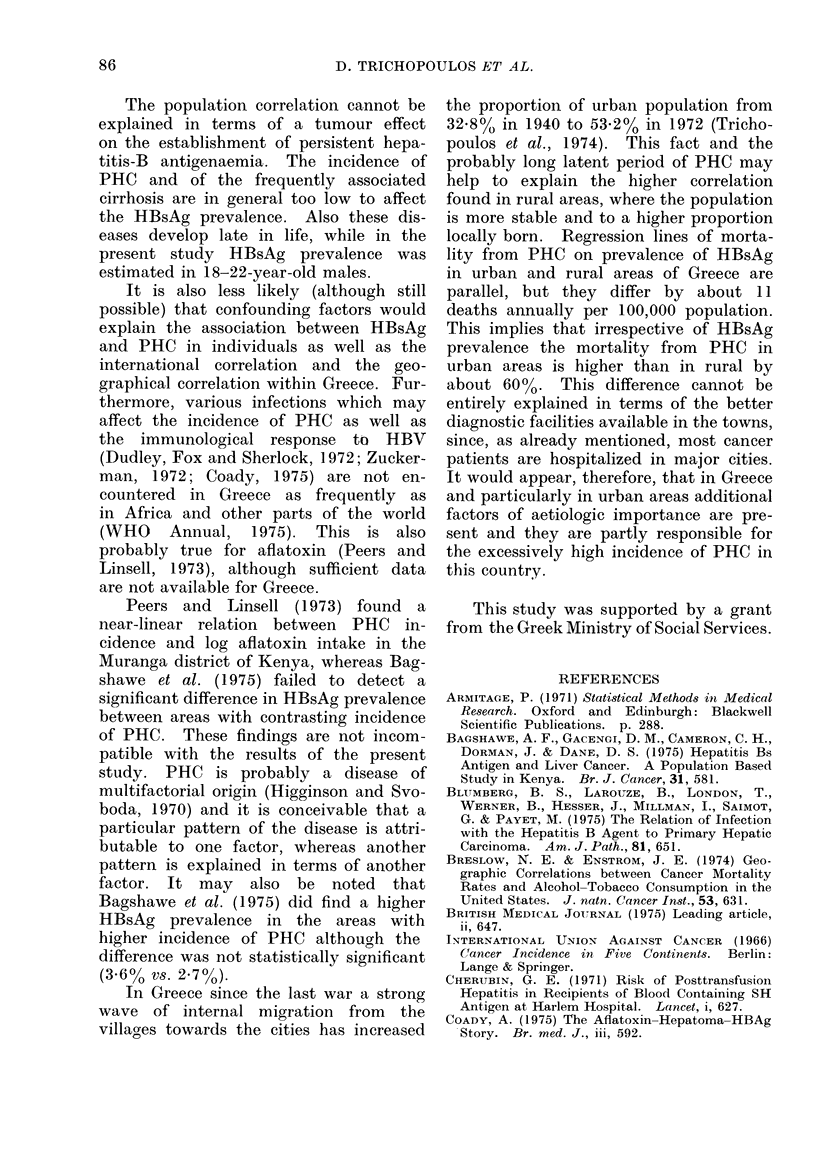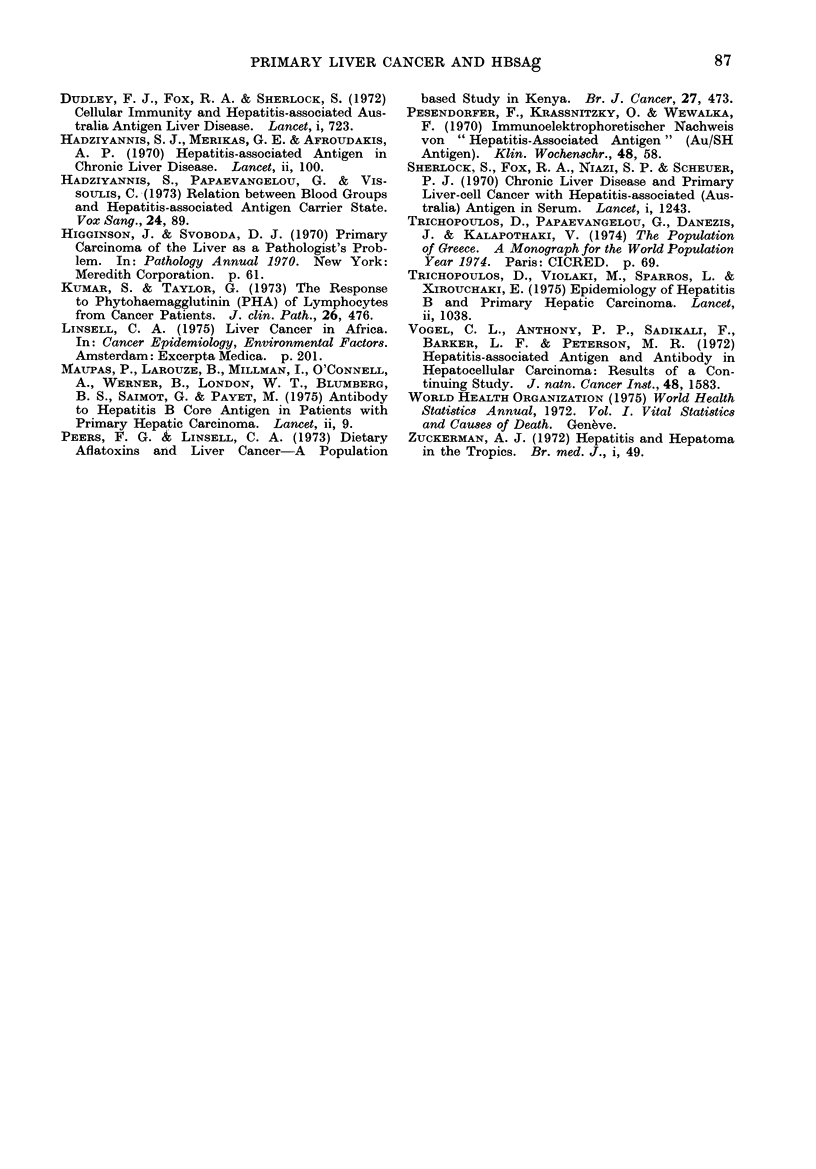# Geographic correlation between mortality from primary hepatic carcinoma and prevalence of hepatitis B surface antigen in Greece.

**DOI:** 10.1038/bjc.1976.125

**Published:** 1976-07

**Authors:** D. Trichopoulos, G. Papaevangelou, M. Violaki, C. Vissoulis, L. Sparros, O. N. Manousos

## Abstract

Average annual age-adjusted mortality rates per 100,000 from primary hepatic carcinoma (PHC) among males for 1971-1973 in the urban and rural areas of the 9 geographical regions of Greece were estimated. Hepatitis-B surface antigen (HBsAg) prevalence by region and area was evaluated in a sample of 22,844 Greek Air Force recruits from all parts of the country. Mortality from PHC was found significantly higher in urban areas (28-30 vs. 18-81) whereas prevalence of HBsAg was higher in rural areas (5-3% vs. 3-90%). Nevertheless further statistical analysis showed that there is a strong correlation between HBsAg prevalence and mortality from PHC, which is higher in rural (r = + 0-88) than in urban (+ 0-57) areas. The latter findings indicate that hepatitis B infection and PHC may be causally related.


*Br. J. Cancer* (1976) **34**, 83

### GEOGRAPHIC CORRELATION BETWEEN MORTALITY FROM PRIMARY HEPATIC CARCINOMA AND PREVALENCE OF HEPATITIS B SURFACE ANTIGEN IN GREECE

D. TRICHOPOULOS, G. PAPAEVANGELOU, M. VIOLAKI, CH. VISSOULIS, L. SPARROS AND O. N. MANOUSOS

*From the Department of Hygiene and Epidemiology, University of Athens Medical School, Greece*

Received 5 February 1976 Accepted 23 March 1976

**Summary.—Average annual age-adjusted mortality rates per 100,000 from primary hepatic carcinoma (PHC) among males for 1971–1973 in the urban and rural areas of the 9 geographical regions of Greece were estimated. Hepatitis-B surface antigen (HBsAg) prevalence by region and area was evaluated in a sample of 22,844 Greek Air Force recruits from all parts of the country. Mortality from PHC was found significantly higher in urban areas (28·30 *vs.* 18·81) whereas prevalence of HBsAg was higher in rural areas (5·37% *vs.* 3·90%). Nevertheless further statistical analysis showed that there is a strong correlation between HBsAg prevalence and mortality from PHC, which is higher in rural (r = + 0·88) than in urban (+ 0·57) areas. The latter findings indicate that hepatitis B infection and PHC may be causally related.**

THE association between hepatitis-B virus (HBV) infection and primary hepatic carcinoma (PHC) is supported by observations on the natural history of chronic liver disease (Sherlock *et al.*, 1970; Hadziyannis, Merikas and Afroudakis, 1970) as well as by findings of studies showing a greater than expected prevalence of hepatitis-B surface antigen (HBsAg) (Vogel *et al.*, 1972; Blumberg *et al.*, 1975) and antibody to hepatitis-B core antigen in patients with PHC (Maupas *et al.*, 1975).

Although this association seems well documented, at least in areas of the world with high incidence of PHC, there is some doubt about the causal nature of the relationship. Indeed the association could be secondary, or the PHC and the frequently related chronic liver disease could facilitate the establishment of persistent antigenaemia (Kumar and Taylor, 1973; Linsell, 1975; Coady, 1975).

Population correlation between prevalence of HBsAg and incidence of PHC could be considered as further evidence of the aetiological significance of HBV infection in the development of PHC. Indeed it was found that the prevalence of HBsAg is high in the general population of areas such as Africa and Greece where PHC is very common (*British Medical Journal*, 1975; Trichopoulos *et al.*, 1975). However, the validity of this international population correlation is questionable, since the compared populations are very different in many aspects and the data concerning the prevalence of HBsAg and the incidence of PHC are not strictly comparable. Clearly geographical correlations based on homogeneous population groups and comparable data would have greater value. It is of interest that there has been only one study of this nature, in the Muranga district of Kenya, based on a relatively small sample, and this has shown no significant difference of HBsAg prevalence

Address for correspondence: Professor D. Trichopoulos, Department of Hygiene and Epidemiology, University of Athens Medical School, Goudi, Athens 609, Greece.

between areas of high and low incidence of PHC (Bagshawe *et al.*, 1975).

The present study was designed to investigate the geographical correlation between HBsAg prevalence and PHC mortality in the various regions of Greece.

### SUBJECTS AND METHODS

*Prevalence of HBsAg.*—Between 1971 and 1975 22,844 Greek Air Force recruits were screened for HBsAg at the time of their enlistment. Their age varied between 18 and 22 years. The recruits were classified according to their permanent residence in urban (cities or towns of more than 10,000 inhabitants) or rural (villages or towns of less than 10,000 inhabitants) areas of the nine main geographical regions of Greece.

Sera were collected aseptically and stored at −20°C until tested. HBsAg was detected by counterimmunoelectrophoresis (Pesendorfer, Krassnitzky and Wewalka, 1970).

*Mortality from PHC.*—Population data from the 1971 census and death certificates for the years 1971, 1972 and 1973, compiled by the health services branch of the Ministry of Social Services, were used to estimate average annual age-specific mortality rates among males from cause 155 of the International Classification of Diseases of the World Health Organization, 1965 revision (malignant neoplasms of liver and intrahepatic bile ducts, specified as primary). Although most PHC cases are treated in major hospitals of the larger cities, deaths are classified according to the place of permanent residence. Annual mortality rates from PHC by geographic region and urban–rural areas within region were age-adjusted to the distribution of the total male population of Greece of the 1971 census (Trichopoulos *et al.*, 1974).

### RESULTS

The prevalence of HBsAg among Greek Air Force recruits was 4·46%. It was significantly higher in rural (5·37%) than in urban (3·90%) areas ($P < 0{\cdot}001$). The average annual mortality rate from PHC per 100,000 male population was 23·12. It was significantly higher in

TABLE.—*Male Population, Average Annual Mortality from PHC among Males* (1971–1973) *and Prevalence of HBsAg among Greek Air Force Recruits* (1971–1975) *by Place of Residence in Urban or Rural Areas of the Nine Geographical Regions of Greece*

| Geographical region | and area | Male population (census 1971) | Mortality* from PHC (per 100,000) | Prevalence of HBsAg: Number examined | Number of carriers | % carriers |
|---|---|---|---|---|---|---|
| Central Greece: | urban | 1,352,680 | 28·26 | 7,770 | 249 | 3·20 |
| | rural | 355,140 | 19·12 | 1,597 | 77 | 4·82 |
| Peloponnesos: | urban | 146,020 | 23·67 | 1,211 | 51 | 4·21 |
| | rural | 345,320 | 15·64 | 1,903 | 66 | 3·47 |
| Ionian Islands: | urban | 15,580 | 27·80 | 78 | 1 | 1·28 |
| | rural | 71,460 | 17·15 | 269 | 8 | 2·97 |
| Epirus: | urban | 36,280 | 26·23 | 280 | 8 | 2·86 |
| | rural | 111,940 | 20·20 | 476 | 22 | 4·62 |
| Thessaly: | urban | 115,540 | 34·89 | 1,049 | 67 | 6·39 |
| | rural | 203,840 | 22·98 | 1,115 | 66 | 5·92 |
| Macedonia: | urban | 433,840 | 29·66 | 2,697 | 144 | 5·34 |
| | rural | 507,580 | 23·51 | 1,853 | 159 | 8·58 |
| Thrace: | urban | 49,300 | 25·31 | 192 | 8 | 4·17 |
| | rural | 113,100 | 23·96 | 409 | 34 | 8·31 |
| Aegean Islands: | urban | 49,400 | 30·15 | 222 | 8 | 3·60 |
| | rural | 151,420 | 12·05 | 572 | 18 | 3·15 |
| Crete: | urban | 75,660 | 21·47 | 615 | 15 | 2·44 |
| | rural | 145,960 | 13·25 | 536 | 17 | 3·17 |
| Greece, Total: | urban | 2,274,300 | 28·30 | 14,164 | 552 | 3·90 |
| | rural | 2,005,760 | 18·81 | 8,680 | 466 | 5·37 |
| Grand Total | | 4,280,060 | 23·12 | 22,844 | 1,018 | 4·46 |

* Rates are age-adjusted to the distribution of the total male population of Greece.

FIG.—Linear regression lines of annual mortality (per 100,000) from PHC on prevalence (per cent) of HBsAg in urban (1) and rural (2) areas of the nine geographical regions of Greece.

urban (28·30) than in rural (18·81) areas ($P < 0{\cdot}001$).

The Table shows the average age-adjusted annual mortality rates from PHC among males in the urban and rural areas of the 9 geographic regions of Greece. It shows also the prevalence of HBsAg among Greek Air Force recruits by place of permanent residence as above.

Covariance analysis (Armitage, 1971) showed that the slopes of the regression lines (Fig.) of mortality from PHC on HBsAg prevalence in urban ($1{\cdot}47 \pm 0{\cdot}80$) and rural ($1{\cdot}79 \pm 0{\cdot}37$) areas were not significantly different ($P > 0{\cdot}40$). The estimated common slope ($b = 1{\cdot}68 \pm 0{\cdot}39$) is significantly different from zero ($P < 0{\cdot}001$).

The correlation coefficient between the two variables is $+0{\cdot}88$ in rural and $+0{\cdot}57$ in urban areas with a common estimate of $+0{\cdot}76$, which is again significantly different from zero ($P < 0{\cdot}001$).

If the urban–rural difference in the prevalence of HBsAg is allowed for, the difference between the mean mortality from PHC in urban and rural areas is further increased. The adjusted difference (Armitage, 1971) in mortality from PHC between urban and rural areas is $10{\cdot}99 \pm 1{\cdot}42$ and differs significantly from zero ($P < 0{\cdot}001$).

### DISCUSSION

The high prevalence of HBsAg in Greece has been noted by several authors (Hadziyannis *et al.*, 1973). It is of interest that this prevalence is higher in rural than in urban areas, a fact which could be attributed to the lower hygiene standards of the rural population (Cherubin, 1971).

The estimated annual mortality rate from PHC is much higher than that prevailing in other European countries (I.U.A.C., 1966).

There are certain limitations in the mortality data. In Greece the majority of PHC cases are diagnosed on clinical grounds, since autopsies are rarely performed and alpha-foetoprotein determinations are not routinely performed in most hospitals. It seems likely however that the possible errors are not related to the regional distribution of HBsAg and this substantially decreases the possibility that the association is not real. Therefore, the present study helps to establish that there is a geographical correlation between HBsAg prevalence and mortality from (and hence incidence of) PHC.

Many problems complicate the interpretation of correlation studies. Difficulties arise from the use of populations as sampling units, the long latent interval for most human cancers, and the presence of multiple aetiologic agents. They have recently been discussed by Breslow and Enstrom (1974). However, the demonstration of a population correlation between HBsAg and PHC helps to eliminate a number of alternative explanations for the association of these two factors in individuals.

The population correlation cannot be explained in terms of a tumour effect on the establishment of persistent hepatitis-B antigenaemia. The incidence of PHC and of the frequently associated cirrhosis are in general too low to affect the HBsAg prevalence. Also these diseases develop late in life, while in the present study HBsAg prevalence was estimated in 18–22-year-old males.

It is also less likely (although still possible) that confounding factors would explain the association between HBsAg and PHC in individuals as well as the international correlation and the geographical correlation within Greece. Furthermore, various infections which may affect the incidence of PHC as well as the immunological response to HBV (Dudley, Fox and Sherlock, 1972; Zuckerman, 1972; Coady, 1975) are not encountered in Greece as frequently as in Africa and other parts of the world (WHO Annual, 1975). This is also probably true for aflatoxin (Peers and Linsell, 1973), although sufficient data are not available for Greece.

Peers and Linsell (1973) found a near-linear relation between PHC incidence and log aflatoxin intake in the Muranga district of Kenya, whereas Bagshawe *et al.* (1975) failed to detect a significant difference in HBsAg prevalence between areas with contrasting incidence of PHC. These findings are not incompatible with the results of the present study. PHC is probably a disease of multifactorial origin (Higginson and Svoboda, 1970) and it is conceivable that a particular pattern of the disease is attributable to one factor, whereas another pattern is explained in terms of another factor. It may also be noted that Bagshawe *et al.* (1975) did find a higher HBsAg prevalence in the areas with higher incidence of PHC although the difference was not statistically significant (3·6% *vs.* 2·7%).

In Greece since the last war a strong wave of internal migration from the villages towards the cities has increased the proportion of urban population from 32·8% in 1940 to 53·2% in 1972 (Trichopoulos *et al.*, 1974). This fact and the probably long latent period of PHC may help to explain the higher correlation found in rural areas, where the population is more stable and to a higher proportion locally born. Regression lines of mortality from PHC on prevalence of HBsAg in urban and rural areas of Greece are parallel, but they differ by about 11 deaths annually per 100,000 population. This implies that irrespective of HBsAg prevalence the mortality from PHC in urban areas is higher than in rural by about 60%. This difference cannot be entirely explained in terms of the better diagnostic facilities available in the towns, since, as already mentioned, most cancer patients are hospitalized in major cities. It would appear, therefore, that in Greece and particularly in urban areas additional factors of aetiologic importance are present and they are partly responsible for the excessively high incidence of PHC in this country.

This study was supported by a grant from the Greek Ministry of Social Services.

### REFERENCES

ARMITAGE, P. (1971) *Statistical Methods in Medical Research.* Oxford and Edinburgh: Blackwell Scientific Publications. p. 288.

BAGSHAWE, A. F., GACENGI, D. M., CAMERON, C. H., DORMAN, J. & DANE, D. S. (1975) Hepatitis Bs Antigen and Liver Cancer. A Population Based Study in Kenya. *Br. J. Cancer*, **31**, 581.

BLUMBERG, B. S., LAROUZE, B., LONDON, T., WERNER, B., HESSER, J., MILLMAN, I., SAIMOT, G. & PAYET, M. (1975) The Relation of Infection with the Hepatitis B Agent to Primary Hepatic Carcinoma. *Am. J. Path.*, **81**, 651.

BRESLOW, N. E. & ENSTROM, J. E. (1974) Geographic Correlations between Cancer Mortality Rates and Alcohol–Tobacco Consumption in the United States. *J. natn. Cancer Inst.*, **53**, 631.

BRITISH MEDICAL JOURNAL (1975) Leading article, ii, 647.

INTERNATIONAL UNION AGAINST CANCER (1966) *Cancer Incidence in Five Continents.* Berlin: Lange & Springer.

CHERUBIN, G. E. (1971) Risk of Posttransfusion Hepatitis in Recipients of Blood Containing SH Antigen at Harlem Hospital. *Lancet*, i, 627.

COADY, A. (1975) The Aflatoxin–Hepatoma–HBAg Story. *Br. med. J.*, iii, 592.

DUDLEY, F. J., FOX, R. A. & SHERLOCK, S. (1972) Cellular Immunity and Hepatitis-associated Australia Antigen Liver Disease. *Lancet*, i, 723.

HADZIYANNIS, S. J., MERIKAS, G. E. & AFROUDAKIS, A. P. (1970) Hepatitis-associated Antigen in Chronic Liver Disease. *Lancet*, ii, 100.

HADZIYANNIS, S., PAPAEVANGELOU, G. & VISSOULIS, C. (1973) Relation between Blood Groups and Hepatitis-associated Antigen Carrier State. *Vox Sang.*, **24**, 89.

HIGGINSON, J. & SVOBODA, D. J. (1970) Primary Carcinoma of the Liver as a Pathologist's Problem. In: *Pathology Annual 1970*. New York: Meredith Corporation. p. 61.

KUMAR, S. & TAYLOR, G. (1973) The Response to Phytohaemagglutinin (PHA) of Lymphocytes from Cancer Patients. *J. clin. Path.*, **26**, 476.

LINSELL, C. A. (1975) Liver Cancer in Africa. In: *Cancer Epidemiology, Environmental Factors*. Amsterdam: Excerpta Medica. p. 201.

MAUPAS, P., LAROUZE, B., MILLMAN, I., O'CONNELL, A., WERNER, B., LONDON, W. T., BLUMBERG, B. S., SAIMOT, G. & PAYET, M. (1975) Antibody to Hepatitis B Core Antigen in Patients with Primary Hepatic Carcinoma. *Lancet*, ii, 9.

PEERS, F. G. & LINSELL, C. A. (1973) Dietary Aflatoxins and Liver Cancer—A Population based Study in Kenya. *Br. J. Cancer*, **27**, 473.

PESENDORFER, F., KRASSNITZKY, O. & WEWALKA, F. (1970) Immunoelektrophoretischer Nachweis von "Hepatitis-Associated Antigen" (Au/SH Antigen). *Klin. Wochenschr.*, **48**, 58.

SHERLOCK, S., FOX, R. A., NIAZI, S. P. & SCHEUER, P. J. (1970) Chronic Liver Disease and Primary Liver-cell Cancer with Hepatitis-associated (Australia) Antigen in Serum. *Lancet*, i, 1243.

TRICHOPOULOS, D., PAPAEVANGELOU, G., DANEZIS, J. & KALAPOTHAKI, V. (1974) *The Population of Greece. A Monograph for the World Population Year 1974*. Paris: CICRED. p. 69.

TRICHOPOULOS, D., VIOLAKI, M., SPARROS, L. & XIROUCHAKI, E. (1975) Epidemiology of Hepatitis B and Primary Hepatic Carcinoma. *Lancet*, ii, 1038.

VOGEL, C. L., ANTHONY, P. P., SADIKALI, F., BARKER, L. F. & PETERSON, M. R. (1972) Hepatitis-associated Antigen and Antibody in Hepatocellular Carcinoma: Results of a Continuing Study. *J. natn. Cancer Inst.*, **48**, 1583.

WORLD HEALTH ORGANIZATION (1975) *World Health Statistics Annual*, 1972. *Vol. I. Vital Statistics and Causes of Death*. Genève.

ZUCKERMAN, A. J. (1972) Hepatitis and Hepatoma in the Tropics. *Br. med. J.*, i, 49.